# Targeting caveolin-1 deficiency in bone marrow derived cells: a new therapeutic window for fibrotic diseases?

**DOI:** 10.3389/fphar.2014.00193

**Published:** 2014-08-22

**Authors:** Eleonora Cianci, Antonio Recchiuti

**Affiliations:** ^1^Department of Medicine and Aging Science, G. d'Annunzio UniversityChieti, Italy; ^2^Center of Excellence on Aging, G. d'Annunzio UniversityChieti, Italy; ^3^Department of Experimental and Clinical Science, G. d'Annunzio UniversityChieti, Italy

**Keywords:** Inflammation, fibrocytes, cell migration, Pharmacology, cytokines/chemokines, cell differentiation, resolution of inflammation

Tissue repair is an essential component of wound healing and resolution of inflammation that, when uncontrolled, can lead to excessive scarring and organ failure (Majno, 1991). Indeed, exaggerated and persistent extracellular matrix (ECM) deposition in tissues is the hallmark of many pathologies linked to non-resolving inflammation, such as asthma, chronic obstructive pulmonary disease, and atherosclerosis (Nathan and Ding, 2010; Serhan et al., 2010; Tabas and Glass, 2013). Systemic fibrosis, also called scleroderma (SSc) is a rare, often idiopathic, autoimmune disease in which a chronic inflammation leads to diffuse fibrosis in several organs, mainly skin, lungs, kidneys, eyes, heart, liver (Shah and Wigley, 2014). Despite considerable advances, this pathology, which affects several million of individuals worldwide, is still without a specific and effective therapy (Canady et al., 2013)

The ancient dogma that fibrosis originates from hyperactivation or deregulation of tissue resident fibroblasts by the local milieu of cytokines and growth factors has been challenged by accruing evidence signifying now that many cells can contribute to the pathogenesis of fibrosis. These include bone marrow (BM)-derived monocytes that can differentiate into “fibrocytes,” i.e., collagen-producing leukocytes (Bucala et al., 1994), that can not only release ECM proteins, but also secrete pro-inflammatory cytokines, chemokines, and growth factors that amplify the pro-fibrotic process in a vicious circle (Metz, 2003). Hence, bone marrow derived fibrocytes represent an important cellular target in SSC and other chronic diseases.

It is well documented that in fibrotic diseases caveolin (Cav)-1 expression is profoundly reduced in fibroblast and monocytes. Cav-1 is a master regulator of several kinases downstream of growth factor, chemokines, and cytokine receptors, as well as many other cellular functions [e.g., ECM adhesion, lipid transport, and membrane traffic (Boscher and Nabi, 2012)]. Lack of Cav-1 in SSc patients and nullified mice results in collagen overexpression and monocyte hypermigration. Therefore, in recent years, Cav-1 has been object of intense scrutiny as possible molecular pharmaceutical target to treat SSc and other fibrotic diseases (Tourkina et al., 2008).

In this Research Topic of Frontiers in Pharmacology named The Cell Types of Fibrosis, Lee et al. (2014) demonstrate that treatment with a Cav-1 scaffolding domain peptide (CSD) spanning amino acids 82–101 of the full protein inhibits dermal fibrosis and lipodystrophy in a mouse model of bleomycin-induced fibrosis that closely mimic the histopathology of skin observed in clinical situations of SSc. Namely, the authors demonstrate that CSD significantly reduced accumulation in skin and dermis of SSc mice of chemokine receptor (CCR) 5 positive monocytes and fibrocytes, which are increased in fibrotic lesions of SSc mice and SSc patients, and ameliorated the clinical signs of skin fibrosis. Moreover, CSD treatment reduced migration of SSc monocytes toward CCR5 ligands and diminished receptor expression in SSc monocytes.

These results represent a step forward toward the exploitation of Cav-1 directed therapeutics for the treatment of SSc and open the road for further studies aimed at establishing the molecular mechanisms by which CSD protects from fibrosis. For instance, additional studies could investigate whether CSD acts by re-establishing the correct turnover of CCR5 upon engagement to its ligands. Or, whether CSD modifies the phenotype of BM-derived fibrocytes (e.g., reducing their capability of secreting ECM components and other pro-fibrotic mediators). Furthermore, the study of Lee and colleagues provides proof of concept for testing CSD for the treatment of fibrosis in different pathologies characterized by a deregulation of monocyte plasticity and functions. For example, in atherosclerotic plaque monocyte-macrophages can either differentiate into lipid rich “foam cells” and secrete factors that sustain atheroma formation or can be stimulated to promote resolution (Moore and Tabas, 2011; Randolph, 2014). Likewise, macrophages accumulate in obese fat tissues (Weisberg et al., 2003) and have a distinct signature of lipid mediators, cytokines, and adipokines in obesity that can be skewed to limit the inflammatory tone (Hellmann et al., 2011; Titos et al., 2011). Finally, lung fibrosis is a hallmark of chronic respiratory pathologies such as asthma, chronic obstructive pulmonary disease, and cystic fibrosis in which airways are repeatedly exposed to insults (Levy and Serhan, 2014). Whether Cav-1 deficiency and increased infiltration of BM-derived fibrocytes also occur in these and other illnesses, and whether CSD treatment proves therapeutically effective is of paramount interest.

## Conflict of interest statement

The authors declare that the research was conducted in the absence of any commercial or financial relationships that could be construed as a potential conflict of interest.
